# Estimation of the genetic milk yield parameters of Holstein cattle under heat stress in South Korea

**DOI:** 10.5713/ajas.18.0258

**Published:** 2018-07-26

**Authors:** SeokHyun Lee, ChangHee Do, YunHo Choy, ChangGwon Dang, Alam Mahboob, Kwanghyun Cho

**Affiliations:** 1 Animal Breeding & Genetics Division, National Institute of Animal Science, RDA, Cheonan 31000, Korea; 2 Division of Animal and Dairy Science, Chungnam National University, Daejeon 34134, Korea; 3 Department of Dairy Science, Korea National College of Agriculture and Fisheries, Jeonju 54874, Korea

**Keywords:** Heat Tolerance, Temperature-humidity Index, Milk Yield, Heritability

## Abstract

**Objective:**

The objective of this study was to investigate the genetic components of daily milk yield and to re-rank bulls in South Korea by estimated breeding value (EBV) under heat stress using the temperature-humidity index (THI).

**Methods:**

This study was conducted using 125,312 monthly test-day records, collected from January 2000 to February 2017 for 19,889 Holstein cows from 647 farms in South Korea. Milk production data were collected from two agencies, the Dairy Cattle Genetic Improvement Center and the Korea Animal Improvement Association, and meteorological data were obtained from 41 regional weather stations using the Automated Surface Observing System (ASOS) installed throughout South Korea. A random regression model using the THI was applied to estimate genetic parameters of heat tolerance based on the test-day records. The model included herd-year-season, calving age, and days-in-milk as fixed effects, as well as heat tolerance as an additive genetic effect, permanent environmental effect, and direct additive and permanent environmental effect.

**Results:**

Below the THI threshold (≤72; no heat stress), the variance in heat tolerance was zero. However, the heat tolerance variance began to increase as THI exceeded the threshold. The covariance between the genetic additive effect and the heat tolerance effect was −0.33. Heritability estimates of milk yield ranged from 0.111 to 0.176 (average: 0.128). Heritability decreased slightly as THI increased, and began to increase at a THI of 79. The predicted bull EBV ranking varied with THI.

**Conclusion:**

We conclude that genetic evaluation using the THI function could be useful for selecting bulls for heat tolerance in South Korea.

## INTRODUCTION

In South Korea, it has become necessary to evaluate dairy bull performance based on quantifiable factors exerting a beyond the Korean Peninsula, because the country has started to export dairy bull semen to countries with distinctly different environments. One such factor is climatic changes, which affect bull performance. Previous studies have suggested that the Earth’s climate has warmed during the past century, and this change has continued at an unprecedented and continuous rate in recent years [1]. These recent climate changes are challenging for the dairy industry because animals in high-temperature environments voluntarily decrease their feed intake to reduce digestive heat production, which in turn causes a reduction in milk yield and a negative energy balance [2–4]. Cows with high milking abilities, in particular, are more susceptible to such heat-stress conditions [5]. These conditions drive dairy breeding schemes toward improvements in genetic predisposition for higher milking ability under stressful conditions.

Nearly two decades ago, a study by Misztal [6] introduced a temperature-humidity index (THI)-based methodology, combining test-day records with weather data collected from public weather stations, for the genetic evaluation of animals with respect to heat tolerance. A clear advantage of using public weather station data is their reliability, because these stations do not rely on individual measurements of body temperature or respiration, which are difficult to generate at large scales. Ravagnolo and Misztal [7] also suggested the use of public weather data for national evaluations. The THI is a bioclimatic index now commonly used to determine the degree of heat stress in studies of animals including dairy cattle [5,8,9]. The THI is a unit measurement that represents a combination of air temperature and humidity [5,9,10]. Generally, the THI function considers three scenarios. First, the daily production (milk yield) of a cow begins to decrease after a THI threshold. Such production drops are typically nonlinear, but can be linearized by the THI function if necessary. Second, there should be genetic variability in susceptibility to heat stress. Dairy production by individual cows may drop at one particular threshold (scenario 1), at various different thresholds (scenario 2), or under both scenarios (scenario 3). There may also be differential individual rates of production decline as indexed by the THI. However, variability in the differential rate of decline (scenario 1) is preferred when using the THI function due to its better theoretical tractability; it is theoretically easier to express than other scenarios. Third, heat stress should comprise both genetic and non-genetic components, where two components are changed by the same curve (THI function) [7].

Therefore, the objective of this study was to investigate the effect of THI on the genetic variance components of daily milk yield and probable re-ranking of bulls in South Korea by estimated breeding value (EBV) under heat stress conditions.

## MATERIALS AND METHODS

### Data

This study was conducted using 125,312 monthly test-day records, collected from January 2000 to February 2017 for 19,889 Holstein cows from 647 farms in South Korea. Milk production data were provided by two agencies: the Dairy Cattle Genetic Improvement Center and the Korea Animal Improvement Association. We excluded records with extreme milk production (<2 or >61 kg), age at calving (<17 or >31 months), days in milk (DIM, <5 or >500 days) and THI (<61 on test-day) from the data set. Milk records for each cow with at least three test-day records on test days when the THI was >72 in each lactation were retained for analysis. The minimum size of each contemporary group (herd-year-season, HYS) was five. Each cow was milked twice per day.

Meteorological data were obtained from 41 regional weather stations with the Automated Surface Observing System (ASOS) installed throughout Korea. The data included the maximum, minimum, and average daily temperature, and average daily humidity records. Weather variables used in this study included daily maximum temperature and daily average humidity. Test-day THI records indicate the heat stress response, according to Ravagnolo et al [7].

The THI was calculated for each day and weather station using the following expression [10]:

THI (T,RH)=(1.8×T+32)-[(0.55-0.0055×RH)×(1.8×T-26.8)]

Where T and RH are the maximum daily temperature (°C) and average relative humidity (%), respectively.

Table 1 shows the distribution of data by calving age, calving season, and THI.

### Statistical analysis

Genetic components of heat stress and other variables were estimated using the following mixed-model equation:

$$y_{ijkl}={HYS}_{i}+{age}_{j}+{DIM}_{k}+a_{l}+f(i)\times v_{l}+p_{l}+f(i)\times q_{l}+e_{ijkl}$$

Where *y_ijkl_* is the milk yield of the *l*th animal of the *i*th herd-year-season, recorded at the *j*th calving age and the *k*th DIM; *HYS_i_* is the effect of the *i*th herd-year-season (*i* = 1 to 2,483); *age_j_* is the effect of the *j*th calving age (*j* = 1 to 2); *DIM_k_* is the effect of the *k*th DIM (*k* = 1 to 50); *a_l_* and *p_l_* are the effects of the additive genetic merit and permanent environment of the cow l (*l* = 1 to 56,132); *f*(*i*) is the heat stress function for the herd-test-day (HTD); *v_l_* and *q_l_* are the additive and permanent environmental effects of heat tolerance on cow l, respectively; and *e_ijkl_* is the residual effect.

The variances are:

$$E\;\left(\begin{array}{c} y\\ a\\ v\\ p\\ q\\ e \end{array}\right)=\left(\begin{array}{c} X\beta \\ 0\\ 0\\ 0\\ 0\\ 0 \end{array}\right)\;\;\;Var\;\left(\begin{array}{c} a\\ v\\ p\\ q\\ e \end{array}\right)=\left(\begin{array}{ccccc} A\sigma_{a}^{2} & A\sigma_{av} & 0 & 0 & 0\\ A\sigma_{av} & A\sigma_{v}^{2} & 0 & 0 & 0\\ 0 & 0 & I\sigma_{p}^{2} & I\sigma_{pq} & 0\\ 0 & 0 & I\sigma_{pq} & I\sigma_{q}^{2} & 0\\ 0 & 0 & 0 & 0 & I\sigma_{e}^{2} \end{array}\right)$$

This model can be used to illustrate the repeatability of the model with random regression of the heat stress function, which determines the effect of heat tolerance by calculating the relative change in production per unit of heat stress. To simplify the calculation, residual variance was assumed to be homogeneous. Our preliminary study showed that milk yield started to decrease at a threshold THI of 72. However, milk protein yield and milk fat yield did not meet the threshold because they continued to decline. Therefore, we considered the THI threshold to be 72 [11].

The heat-stress function was defined as [7]:

$$\text{f}(\text{i})=\left(\begin{array}{cc} THI(T,RH)-72 & if\;THI(T,RH)>72\;(THI\;72\;is\;threshold\;point)\\ 0 & Otherwise\;(no\;heat\;stress) \end{array}\right).$$

The variance components were estimated using the EM-REML algorithm in the REMLF90 software module from the BLUPF90 suite of programs [12].

The heritability value used to determine production at heat stress level *f*(*i*) was calculated as:

$$h_{f(i)}^{2}=\frac{\sigma_{a}^{2}+f{\left(i\right)}^{2}\sigma_{v}^{2}+2f(i)\sigma_{av}}{\sigma_{a}^{2}+f{\left(i\right)}^{2}\sigma_{v}^{2}+2f(i)\sigma_{av}+\sigma_{p}^{2}+f{\left(i\right)}^{2}\sigma_{q}^{2}+2f(i)\sigma_{pq}+\sigma_{e}^{2}}.$$

The genetic correlation between the genetic and heat stress effects was calculated as:

$$r_{\text{a},\text{f}(\text{i})\text{v}}=\frac{f(i)\sigma_{a}\sigma_{v}}{\sqrt{\sigma_{a}^{2}+f{\left(i\right)}^{2}\sigma_{v}^{2}}}.$$

## RESULTS AND DISCUSSION

**Figure 1:**
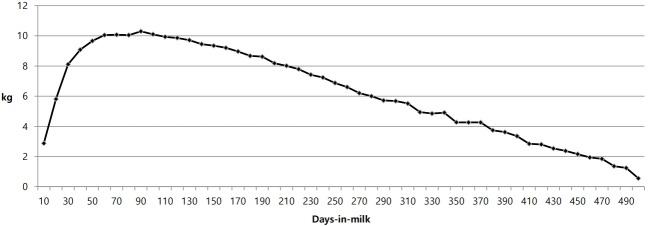
Least squares mean of daily milk yield by days-in-milk.

Figure 1 shows the least-square solutions for DIM classes. These solutions tend to increase before the peak day class (DIM >90) and then decrease. The shapes of the least-squares curves for the DIM classes were similar to a normal lactation curve. In genetic evaluations, test-day records may cause over- or underestimation of parameter estimates due to the different scales of DIM records. Therefore, the test-day model must incorporate the general shape of the lactation curve [13]. Cows exhibit different DIM effects on the same test days. Test-day evaluation results were closely correlated (*r* = 0.87–0.97) to the 305-day evaluation in a previous study [13]. Although a sophisticated test-day model, i.e., a random regression model of the lactation curve, can capture heat stress variation throughout the lactation period, this method has much greater difficulty in estimating the parameters [7]. Therefore, the simple test-day model is more appropriate than the sophisticated test-day model.

The variance component estimates of milk yield are presented in Table 2. The THI values indicate that the variance in genetic heat tolerance was small. The covariance between the genetic additive and heat tolerance effects (*r_a,v_*) was −0.33, indicating that the selecting animals for high milk yield could produce animals susceptible to heat stress. According to a previous study, the total body heat load of lactating cows increased according to the metabolic heat production associated with milk production [14], in turn increasing the ability to maintain homeothermy under heat stress conditions [15]. Other studies have reported correlation coefficients of −0.33 [7] and −0.38 [5], and other similar values [16,17].

**Figure 2:**
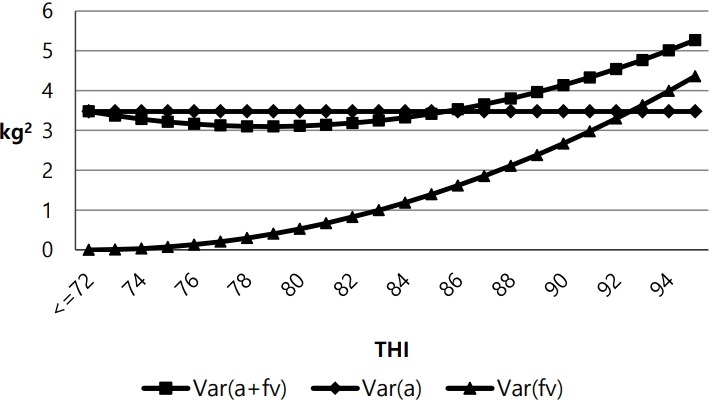
Changes in the estimates of variance components by temperature-humidity index (THI). Additive genetic effects (a, ♦), heat tolerance (fv, ▲), and additive genetic effects plus heat tolerance (a+fv, ■).

Figure 2 shows estimates of additive genetic variance and heat tolerance variance with increasing THI. Under the THI threshold (≤72, no heat stress), the variance in heat tolerance was zero. However, the heat tolerance variance began to increase as THI exceeded the threshold. The total variance was smaller than the genetic variance at THI <86 due to the influence of negative covariance. These findings are similar to those of previous studies [5,7,17,18].

**Figure 3:**
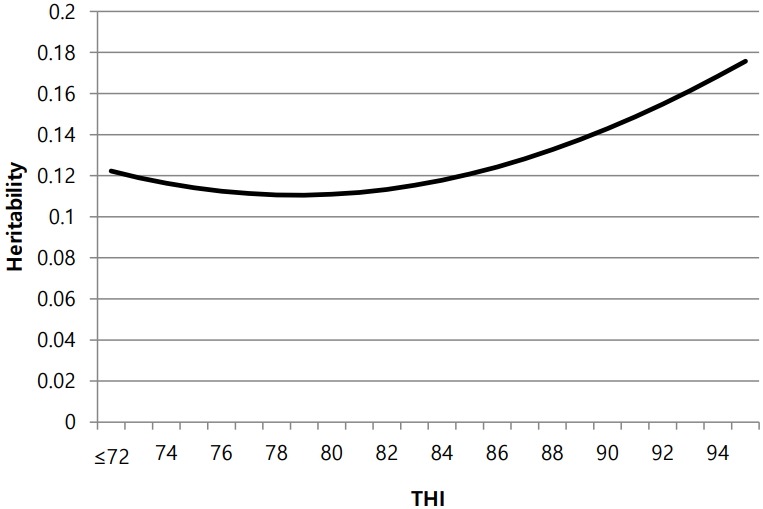
Heritability estimates for milk yield at different temperature-humidity index (THI) values.

Figure 3 shows estimates of heritability for milk yield at different THI values. These estimates ranged from 0.111 to 0.176 (average: 0.128). Heritability decreased slightly as THI increased, and then began to increase at a THI of 79. However, some of these changes may not have been biological, instead being consequences of the random regression model of the THI function [7]. Our estimates were lower than those reported in previous studies that applied the same model (Heritability ranges have been reported as 0.10 to 0.25 by Aguilar et al [19] and 0.16 to 0.21 by Ravagnolo and Misztal [16]) [5,7,17], suggesting that the characteristics of the South Korean Holstein population influence heritability. Table 3 shows the inbreeding coefficient for the data used in this study. Generally, heritability decreased slightly due to increased inbreeding. The South Korean dairy population is small, at nearly 400,000, with a high average inbreeding coefficient of approximately 2.03% in 2012 [19]. The average inbreeding coefficient in this study was 1.5%, and 2,355 of the 56,441 head in pedigree were sires, a smaller population size and higher average inbreeding coefficient than used in previous studies. In the test-day model, HTD is a more suitable variable than herd-year-season. However, the Holstein cattle population in South Korea is small; therefore, we cannot use HTD as a variable because the contemporary group size was insufficient due to the small dairy population size. These differences would lead to larger residual variance than reported in other studies. Hence, our heritability value was lower than that reported elsewhere.

**Figure 4:**
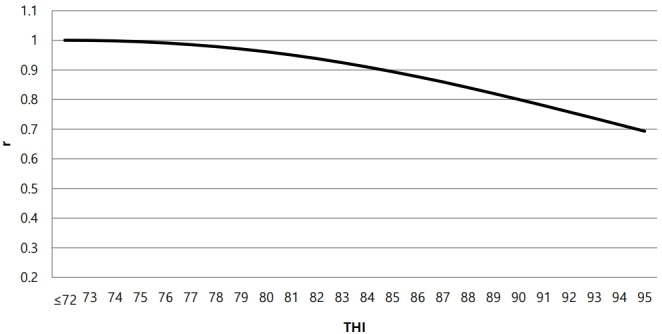
Genetic correlation between breeding values at temperature-humidity index (THI) ≤72 (no heat stress) and breeding values at a given THI for milk yields.

Heat tolerance is among the most important adaptive traits in cattle [5,20,21]. If bulls have similar EBVs, we should consider the effect of heat tolerance on EBV when temperatures are high. Figure 4 shows the genetic correlation between predicted breeding values at a THI ≤72 (no heat stress) and breeding values at a given THI for milk yields. Below the heat stress threshold, the correlation was 1 and started to decrease as THI increased, such that the bull EBV ranking differed according to THI. Table 4 shows the top 10 bulls, having at least 50 daughters, with respect to the EBV for milk yield at different THI values. One bull had negative heat tolerance, and its EBV for milk yield exhibited a greater decrease as THI increased. Bull s1 had the highest EBV for milk yield at THI = 0 (no heat stress). However, bull s1 ranked fourth, and bull s4 ranked first, at THI = 80, because these bulls had a negative and positive heat tolerance EBV, respectively. When heat stress is a factor influencing daily milk yields, selection for high productivity would be useful only if these animals are able to maintain high productivity under heat stress [5,22]. Bull s1 is a good example of this type of condition, because its ranking decreased slightly under heat stress. The heat stress function used meteorological data collected from public weather stations, rather than directly from farms; however, genetic evaluation should not be affected by this substitution, because the use of HYS compensates for farm effects.

## CONCLUSION

We conclude that genetic evaluation using THI could be applied to select bulls in South Korea for thermotolerance. Because daily milk yield and heat tolerance are negatively correlated, selection for high milk production could decrease thermotolerance. Therefore, sire rankings should be changed to incorporate the effects of high temperature and humidity. Our results will be helpful when South Korean dairy cattle are adapting to environmental conditions in other countries, or are selected in a changing climate.

## Figures and Tables

**Table 1:** Distribution, mean, and standard deviation of milk by calving season, calving age, and temperature-humidity index >72

Variable	Class	Observations	%	Mean (kg)	SD (kg)
Calving season	Apr.–Aug.	53,159	42.42	29.47	6.77
	et al.	72,153	57.58	30.13	6.60
Calving age (months)	17–24	56,252	44.89	29.58	6.53
	25–30	69,060	55.11	30.06	6.80
Temperature-humidity index	≤ 72	39,733	31.71	29.68	6.71
	73	4,733	3.78	29.57	6.69
	74	5,934	4.74	29.80	6.84
	75	6,228	4.97	29.80	6.59
	76	5,661	4.52	30.04	6.81
	77	6,934	5.53	29.86	6.61
	78	6,903	5.51	30.23	6.66
	79	6,904	5.51	30.11	6.54
	80	6,993	5.58	29.96	6.60
	81	6,119	4.88	30.33	6.58
	82	5,845	4.66	30.15	6.83
	83	4,770	3.81	29.84	6.59
	84	3,941	3.14	30.10	6.91
	85	3,194	2.55	29.70	6.50
	86	3,172	2.53	30.14	6.86
	87	2,457	1.96	29.33	6.65
	88	2,404	1.92	29.72	6.57
	89	1,430	1.14	29.26	6.44
	90	1,189	0.95	29.24	6.32
	91	454	0.36	29.20	5.89
	92	127	0.10	29.42	4.32
	93	119	0.09	26.34	6.17
	94	27	0.02	28.97	4.75
	95	41	0.03	26.96	5.33
TOTAL		125,312	100	29.85	6.68

SD, standard deviation.

**Table 2:** Parameters estimated ((co)variances±standard error)

Parameter1)	Milk yield (kg)
$\sigma_{a}^{2}$	3.475±0.3487
*σ_av_*	−0.056±0.0109
$\sigma_{v}^{2}$	0.008±0.0005
$\sigma_{e}^{2}$	12.610±0.0265
$\sigma_{p}^{2}$	12.340±0.2621
*σ_pq_*	−0.029±0.0095
$\sigma_{q}^{2}$	0.001±0.0006
*r_a,v_*	−0.33

1) Subscripts ‘a’ and ‘v’ are additive genetic effects on milk yield and heat tolerance. Subscripts ‘p’ and ‘q’ are permanent environmental effects on milk yield and heat tolerance.

**Table 3:** Average inbreeding coefficients for the studied pedigree

Inbreeding rate (%)	N	%
0	26,038	46.13
0–6.25	29,368	52.03
6.25–12.50	861	1.53
12.50–18.75	53	0.09
18.75–25.00	4	0.01
>25.00	117	0.21
Average		1.46
Total	56,441	

**Table 4:** Estimated breeding value (EBV) and ranking for milk yield of the bulls having at least 50 daughters at different temperature-humidity index values

Sire	EBV (72)	Rank (72)	EBV (73)	Rank (73)	EBV (74)	Rank (74)	EBV (75)	Rank (75)	EBV (76)	Rank (76)	EBV (77)	Rank (77)	EBV (78)	Rank (78)	EBV (79)	Rank (79)	EBV (80)	Rank (80)
S1	4.58	1	4.53	1	4.48	1	4.43	1	4.38	1	4.33	1	4.28	1	4.23	1	4.18	2
S2	4.04	2	4.01	2	3.97	2	3.94	2	3.91	2	3.87	3	3.84	3	3.81	3	3.78	3
S3	3.69	3	3.67	3	3.66	4	3.65	4	3.64	4	3.63	4	3.61	4	3.60	4	3.59	4
S4	3.58	4	3.66	4	3.74	3	3.82	3	3.89	3	3.97	2	4.05	2	4.13	2	4.21	1
S5	3.39	5	3.34	5	3.29	6	3.23	6	3.18	7	3.13	7	3.08	8	3.03	8	2.97	8
S6	3.32	6	3.33	6	3.34	5	3.35	5	3.35	5	3.36	5	3.37	5	3.38	6	3.39	6
S7	3.19	7	3.14	7	3.09	8	3.04	9	2.99	9	2.94	9	2.89	10	2.84	10	2.79	10
S8	3.12	8	3.12	8	3.12	7	3.12	8	3.12	8	3.12	8	3.12	7	3.12	7	3.12	7
S9	3.02	9	3.00	10	2.99	10	2.97	10	2.95	10	2.93	10	2.91	9	2.90	9	2.88	9
S10	2.97	10	2.91	11	2.85	11	2.79	12	2.73	12	2.67	12	2.61	13	2.55	15	2.49	17

## References

[1] IPCC (2014). Climate change 2014: impacts, adaptation, and vulnerability.

[2] West J, Mullinix B, Bernard J (2003). Effects of hot, humid weather on milk temperature, dry matter intake, and milk yield of lactating dairy cows. J Dairy Sci.

[3] Mader TL, Davis M, Brown-Brandl T (2006). Environmental factors influencing heat stress in feedlot cattle. J Anim Sci.

[4] Könyves T, Zlatković N, Memiši N (2017). Relationship of temperature-humidity index with milk production and feed intake of holstein-frisian cows in different year seasons. Wetchasan Sattawaphaet.

[5] Bernabucci U, Biffani S, Buggiotti L (2014). The effects of heat stress in Italian Holstein dairy cattle. J Dairy Sci.

[6] Misztal I (1999). Model to study genetic component of heat stress in dairy cattle using national data. J Dairy Sci.

[7] Ravagnolo O, Misztal I (2000). Genetic component of heat stress in dairy cattle, parameter estimation. J Dairy Sci.

[8] Hahn G, Mader T, Eigenberg R (2003). Perspective on development of thermal indices for animal studies and management. EAAP Technic Ser.

[9] Armstrong D (1994). Heat stress interaction with shade and cooling. J Dairy Sci.

[10] NRC (1971). A guide to environmental research on animals.

[11] Lee S, Choy Y, Dang C-G, Mahboob A, Cho K (2018). Study on the effect of heat stress in milk production traits of Korean Holstein cows. J Korean Data Inf Sci Soc.

[12] Misztal I, Tsuruta S, Strabel T (2002). BLUPF90 and related programs (BGF90).

[13] Ptak E, Schaeffer L (1993). Use of test day yields for genetic evaluation of dairy sires and cows. Livest Prod Sci.

[14] Nardone A, Ronchi B, Lacetera N, Ranieri MS, Bernabucci U (2010). Effects of climate changes on animal production and sustainability of livestock systems. Livest Sci.

[15] Kadzere C, Murphy M, Silanikove N, Maltz E (2002). Heat stress in lactating dairy cows: a review. Livest Sci.

[16] Ravagnolo O, Misztal I (2002). Studies on genetics of heat tolerance in dairy cattle with reduced weather information via cluster analysis. J Dairy Sci.

[17] Boonkum W, Duangjinda M (2015). Estimation of genetic parameters for heat stress, including dominance gene effects, on milk yield in Thai Holstein dairy cattle. Anim Sci J.

[18] Bohmanova J, Misztal I, Tsuruta S, Norman H, Lawlor T (2005). National genetic evaluation of milk yield for heat tolerance of United States Holsteins. Interbull Bull.

[19] Aguilar I, Misztal I, Tsuruta S (2009). Genetic components of heat stress for dairy cattle with multiple lactations. J Dairy Sci.

[20] Won JI, Dang CG, Lim HJ (2016). Analysis of pedigree structure and inbreeding coefficient for performance tested Holstein cows in Korea. J Agric Life Sci.

[21] McManus C, Prescott E, Paludo G (2009). Heat tolerance in naturalized Brazilian cattle breeds. Livest Sci.

[22] Nardone A, Valentini A (2000). The genetic improvement of dairy cows in warm climates.

